# Life changing response to successive surgical interventions on cranial venous outflow: A case report on chronic fatigue syndrome

**DOI:** 10.3389/fneur.2023.1127702

**Published:** 2023-03-30

**Authors:** J. Nicholas P. Higgins, Patrick R. Axon, Andrew M. L. Lever

**Affiliations:** ^1^Department of Radiology, Addenbrooke's Hospital, Cambridge, United Kingdom; ^2^Skull Base Unit, Addenbrooke's Hospital, Cambridge, United Kingdom; ^3^Department of Medicine, University of Cambridge, Cambridge, United Kingdom; ^4^Deparment of Infectious Diseases, Addenbrooke's Hospital, Cambridge, United Kingdom

**Keywords:** chronic fatigue syndrome, fibromyalgia, idiopathic intracranial hypertension, spontaneous intracranial hypotension, cranial venous outflow obstruction

## Abstract

Recognition of similarities between chronic fatigue syndrome and idiopathic intracranial hypertension (IIH) has raised suggestions that they might be connected, with chronic fatigue syndrome representing a mild version of IIH, sharing many of its symptoms, but without the signature features of elevated intracranial pressure that characterize the complete syndrome. A further development of this idea factors in the effects of a cerebrospinal fluid leak, a known complication of IIH, to explain cases where symptoms seem out of proportion to the apparent physiological disturbance. Cranial venous outflow obstruction has been proposed as the pathological substrate. We describe a patient with multiple symptoms, including headache and disabling fatigue, in which this model guided investigation and treatment. Specifically, CT and catheter venography identified focal narrowings of both jugular and the left brachiocephalic veins. Treatment of brachiocephalic obstruction was not feasible. However, in separate surgical procedures, relief of jugular venous obstruction produced incremental and significant clinical improvements which have proven durable over the length of follow-up. We suggest that investigating chronic fatigue syndrome under this model might not only bring benefit to individual patients but also will provide new insights into IIH and its relationship with spontaneous intracranial hypotension.

## Introduction

The recognition of similarities between chronic fatigue syndrome and idiopathic intracranial hypertension (IIH) has raised suggestions that they might be connected, with chronic fatigue syndrome perhaps representing a mild version of IIH, sharing many of its symptoms, but without the signature features of increased intracranial pressure that characterize the complete syndrome (1). One group has shown that patients with chronic fatigue syndrome, on average, have intracranial pressures in the high normal range but with some unequivocally passing the threshold for IIH (2, 3). Another group has reported similar findings in fibromyalgia (4). Both found that reducing intracranial pressure by withdrawing cerebrospinal fluid (CSF) gave symptomatic improvement even when pressure was within normal limits. A third group has found MRI evidence of raised intracranial pressure in many patients with chronic fatigue syndrome (5).

This relatively simple hypothesis, however, does not explain instances of chronic fatigue syndrome in which the level of disability seems far out of proportion to any conceivable notion of a “forme fruste” version of IIH, even if the risk of visual loss is absent. IIH, however, can be complicated by a CSF leak (6–8). Indeed, it is probable that many cases of spontaneous CSF leaks, manifesting as the syndrome of spontaneous intracranial hypotension, have IIH as their underlying condition (6–8). As pertains to IIH without papilloedema, most cases of spontaneous intracranial hypotension also present no clinical signs. Moreover, the distinctive radiological features of low intracranial pressure may be absent, and the debilitating symptoms that can accompany this syndrome might not include the characteristic orthostatic headache (9, 10).

Many of the complex symptoms described in spontaneous intracranial hypotension are also seen in severe cases of chronic fatigue syndrome (11, 12), and recently, a modification of the IIH/chronic fatigue hypothesis has been published to allow for the possibility of a CSF leak (13). In this modification, chronic fatigue syndrome represents a form of IIH that may be mild because the underlying pathology is mild, or a form in which the underlying pathology is more severe, but whose outward manifestation is modified, for better or worse, by the presence of a CSF leak. In either case, papilloedema would be absent.

Intracranial venous hypertension has been proposed as the final common pathway in the development of IIH (14, 15). We describe a patient with disabling chronic fatigue syndrome in whom this disease model guided investigation and treatment.

## Case history

A 46-year-old woman, a partner in a medical practice, with a long history of headache, dizziness, and fatigue had been unable to work for 3 years since waking up one morning with florid positional vertigo and an inability to hold objects steady in her visual field during movement (later diagnosed as oscillopsia). Since that time, she had suffered daily bouts of dizziness, loss of balance, light-headedness, nausea, bowel disturbance, noise and light sensitivity, increased headaches, and often overwhelming fatigue. Other idiosyncratic symptoms were episodes of daytime confusion and sudden feelings of intense thirst. She had two young children and recalled that she had been unable to work during either of her pregnancies mainly because of vomiting.

Her only significant history was of a bicycle accident 25 years previously. She was told that she had a seizure at the scene but was not admitted to hospital until a week later, by that time suffering with headaches, drowsiness, and confusion. A CT scan revealed a left temporal lobe contusion which was treated conservatively. Anticonvulsants were not prescribed. Ever since, however, she had experienced frequent dizzy spells, light-headedness on standing, and episodes of nausea, as well as vague neurological symptoms such as problems with orientation, making it difficult for her to find her way around buildings. She had a single further seizure 3 years later. Again, anticonvulsant medication was not prescribed.

Over the years, she had sought advice from multiple medical specialists. She had received diagnoses of vestibular migraine and mild autonomic dysfunction, with the latter following a tilt table test. MRI brain was normal. Treatments included amitriptyline, topiramate, propranolol, verapamil, botulinum toxin, venlafaxine, pizotifen, vitamin B2, and magnesium and also physiotherapy, cognitive behavioral therapy, exercise therapy, and dietary and lifestyle adjustment.

Clinical examination was normal; her body mass index value was 23 (normal 18–25). There was no papilloedema. All laboratory investigations were normal. Therefore, satisfying the clinical criteria, she was diagnosed with chronic fatigue syndrome and, in accordance with protocols being developed at the time at our institution (2, 3), was put forward for investigation of intracranial pressure.

## Investigation of intracranial pressure and cranial venous outflow

### CT and CT venography

**Figure 1:**
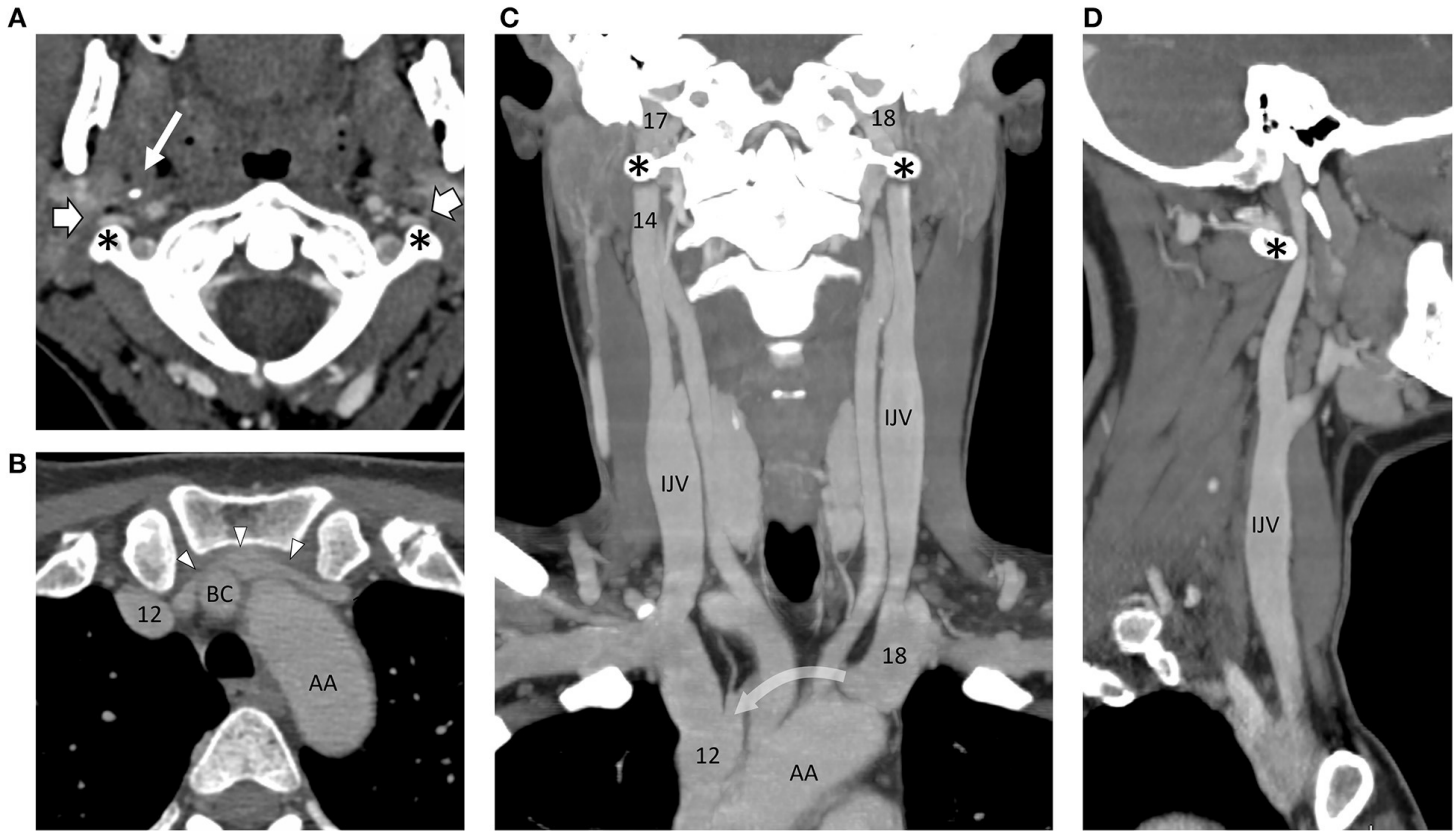
Montage of CT images following intravenous contrast, outlining the arterial and venous angioarchitecture of the neck. **(A)** Axial slice through the C1 vertebra and odontoid peg show the narrowing and flattening of both internal jugular veins (block arrows) as they pass in front of the transverse processes of C1 (asterisks) on either side. The styloid process of the skull (thin arrow) can be seen on the right side. The internal carotid arteries are round in cross-section and situated more medially. **(B)** Axial slice through the thoracic inlet at the level of the left brachiocephalic vein (arrowheads) showing marked narrowing in front of the right brachiocephalic artery (BC, right brachiocephalic artery; AA, aortic arch). **(C)** In this coronal reconstruction, the internal jugular veins (IJVs) lie on the lateral side of the carotid arteries. They are out of the plane as they pass over the C1 transverse processes, but on the sagittal reconstruction **(D)**, the distortion produced on the left side is evident (curved arrow = path of the left brachiocephalic vein, out of the plane in front of the left common carotid and right brachiocephalic arteries.). Numbers are transposed from pressures (cm H_2_O) recorded on catheter venography.

CT brain was normal. CT venography revealed normal intracranial venous sinuses. However, there was striking narrowing of both internal jugular veins anterior to the transverse processes of the C1 vertebra and marked narrowing of the left brachiocephalic vein between the sternum and the origins of the left common carotid and right brachiocephalic arteries (Figure 1).

### Catheter venography and venoplasty

Through a microcatheter, with the patient awake, contrast injections were used to assess the pattern of cranial venous outflow and intraluminal pressure recordings of the significance of any venous narrowing. Venoplasty was used to assess the clinical significance of extracranial venous narrowings by dilating the narrowed segment and monitoring any change in symptoms. These procedures in patients with chronic fatigue syndrome have been described previously (16).

**Figure 2:**
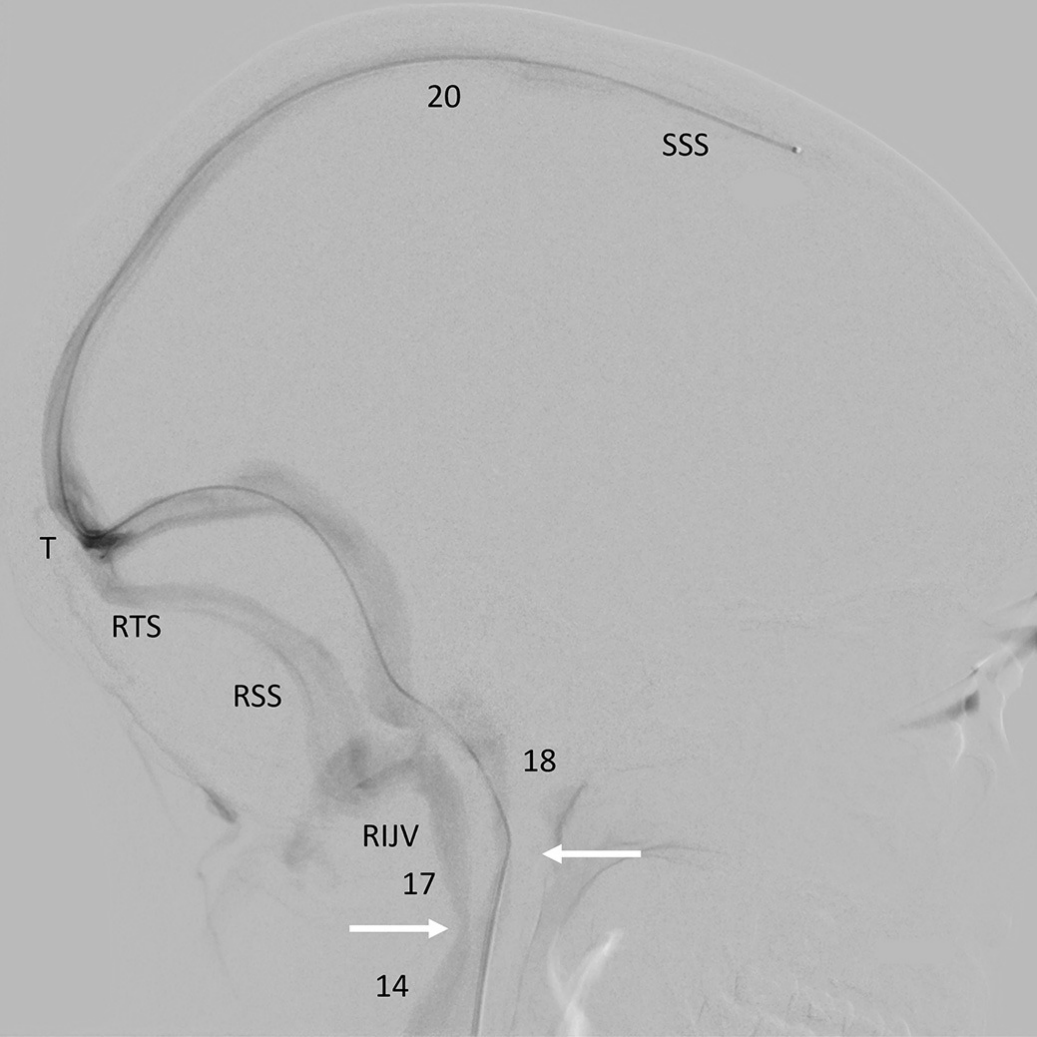
Catheter venogram. Background-subtracted, oblique lateral view of the head and upper neck. Radiographic contrast injected through a catheter passed up through the left lateral sinus into the sagittal sinus outlines the sagittal, both lateral sinuses and upper jugular veins. There is a narrowing of both internal jugular veins (arrows) where they pass over the transverse processes of C1 (SSS, superior sagittal sinus; T, torcula; RTS, right transverse sinus; RSS, right sigmoid sinus; RIJV, right internal jugular vein) (Numbers = pressure recordings in cm H_2_O).

There was mild intracranial venous hypertension (midsagittal sinus pressure = 20 cm H_2_O), with an 8 cm H_2_O pressure gradient between the midsagittal sinus and the superior vena cava, including a 3 cm H_2_O focal gradient across the right jugular narrowing and a 6 cm H_2_O gradient across the tandem narrowings in the left brachiocephalic vein. There was no appreciable gradient across the narrowing of the left upper jugular vein (Figure 2).

The right jugular narrowing was dilated to 8 mm. There was no immediate change but for a week afterward, headache and dizziness were absent, and noise and light intolerance were improved.

She came back subsequently for left brachiocephalic venoplasty, having no symptoms at the time of the procedure but still describing an immediate “clearing” of her head, this benefit lasting until the evening, and then wearing off completely over the next 2 days.

### Lumbar puncture

One month later, lumbar puncture revealed an opening pressure of 14 cm H_2_O. She was not particularly symptomatic at the time, and withdrawal of 17 ml of CSF had no immediate effect, and over the next few days, she had quite severe postural headaches, although commented that when she lay flat in bed, she felt unusually well. As her postural headaches receded, she had 2 days when fatigue and headache were absent, before her usual symptoms returned.

## Diagnostic formulation and first surgery—Right jugular decompression

On the strength of these investigations, she was diagnosed with cranial venous outflow obstruction, with the key lesions being the narrowing of the right jugular vein at the C1 level and of the left brachiocephalic vein anterior to the origins of the great vessels. The significance of left jugular narrowing was uncertain, given the severity of brachiocephalic narrowing downstream.

She was offered decompression of the right jugular vein by the styloidectomy and resection of the right transverse process of C1. Decompression of the brachiocephalic vein was ruled out by the expected difficulty of the procedure, given the uncertainty of the outcome. She was counseled that even if the diagnostic formulation was correct, this surgery would leave the other sites of venous compromise untreated and, therefore, might only be a partial solution to her clinical problem. She had the surgery 16 months after her first consultation. The surgical technique has been described previously (17, 18).

## Progress

Immediately after surgery, her oscillopsia resolved and has not returned. Fatigue, headaches, noise sensitivity, and brain fog were improved. Once home, for example, she found that she was able to do light gardening for 40 min instead of 20. Though she still needed to rest afterward, she was able to finish a full supermarket shop, where previously headache and vertigo routinely forced her to give up halfway, leaving her ill for the rest of the day. She had no further episodes of unexpected thirst.

**Figure 3:**
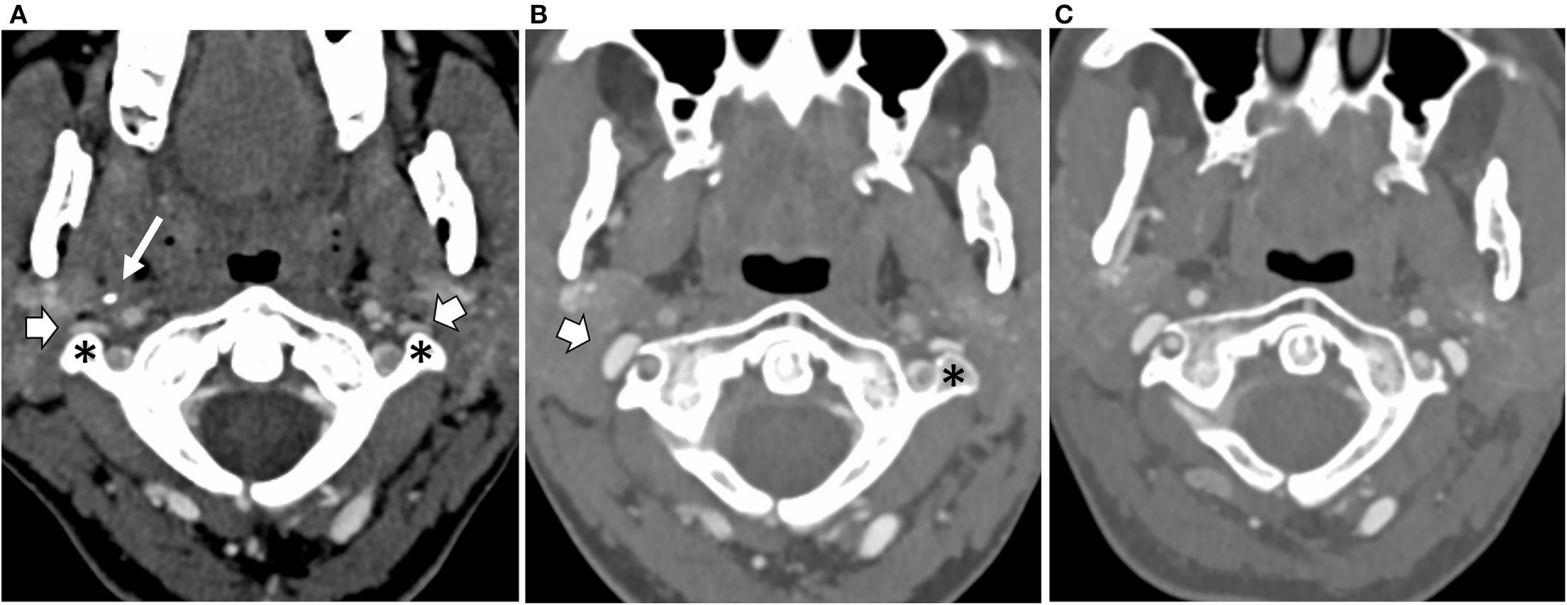
Axial CT scans at the level of the C1 vertebra and odontoid peg following intravenous contrast injection, **(A)** at the baseline, **(B)** following resection of the right styloid and C1 transverse processes, and **(C)** following resection of the left C1 transverse process. The baseline scan **(A)** shows narrowing and flattening of both internal jugular veins (block arrows) as they pass in front of the transverse processes of C1 (asterisk) on either side. The styloid process of the skull (thin arrow) can be seen on the right side. **(B)** Following the right transverse process and styloid resections, the right internal jugular vein (block arrow) has expanded into the space created by the surgery. **(C)** Following left transverse process resection, the left internal jugular vein has now also expanded.

With symptoms stable 12 months post-surgery, repeat CT venography confirmed wide expansion of the right jugular vein at the site of surgical resection. On the left side, jugular and brachiocephalic vein narrowings were unchanged (Figure 3). Limited catheter venography on this occasion revealed a 1 cm H_2_O pressure gradient across the left jugular narrowing and a 4 cm H_2_O gradient along the left brachiocephalic vein. She had left jugular venoplasty, following which her head felt generally better for about 90 min.

## Second surgery—Left jugular decompression

The nature of the brachiocephalic narrowing still precluded surgery. However, her response to venoplasty suggested that there might yet be a benefit in relieving left jugular narrowing, and 2 years following her first surgical procedure she had the second, specifically, decompression of the left jugular vein by resection of the left C1 transverse process, leaving the styloid intact (Figure 3).

## Further progress

There was no change at first, but over the following weeks, her symptoms gradually improved. Fatigue was greatly improved: she could be more active during the day, needing to sleep only one or two afternoons in the week instead of five. She no longer suffered from nausea or diarrhea. Noise sensitivity was improved. Her head was clearer. Headaches were fewer and less severe. She mentioned that previously she had struggled sitting or standing for long periods and that this was greatly improved. She noticed that her resting pulse rate, which had been in the 90 s before treatment, had fallen into the 80 s after her first procedure and was down into the 70 s after her second procedure. Prior to treatment, she had noticed her heart rate increasing in the upright position (confirmed as 18 beats per minute in the tilt table test). Later, she was able to record this increase as consistently reduced to six beats per minute. At 10 months post second surgery, she has been planning a return to work.

## Discussion

The volume and complexity of symptoms experienced by patients with chronic fatigue syndrome, the complete absence of physical signs of ill health, and the complete absence of confirmatory diagnostic tests have placed this condition firmly in the realms of psychological illness in the minds of the majority of medical practitioners (19). Yet similarities with IIH abound, the latter an unequivocally organic condition, the presence of which is only betrayed by signs of raised intracranial pressure, usually papilloedema. Thus, headache and fatigue are usual in both, along with multiple other symptoms, including anxiety, dizziness, depression, body pains, and cognitive and memory disturbance (20–22). Papilloedema, however, is not inevitable, and some patients with IIH do not have any signs of raised intracranial pressure at all (23). These cases are thought to be rare, but in reality, the correct diagnosis depends on clinical suspicion precipitating a diagnostic lumbar puncture, and clinical suspicion is low in the absence of papilloedema (1). Their diagnoses, therefore, if they are given one, will depend on the relative prominence of any one of the multiple other symptoms seen in IIH, and the underlying fact of raised intracranial pressure will be missed.

This idea has been taken up by two independent research groups, one with respect to chronic fatigue syndrome, the other with respect to fibromyalgia, each reporting similar results, specifically that a significant minority of these patients have intracranial pressures above the threshold for IIH and that the majority of those who do not, respond to CSF withdrawal in exactly the same way as those who do (2–4). This implies not only that IIH is being missed but also that the different conditions are connected, with chronic fatigue or fibromyalgia essentially representing IIH in mild form.

If this is correct, then debate regarding the etiology of IIH is relevant to chronic fatigue, and, concerning this, three distinct etiopathological mechanisms have been proposed for IIH: idiopathic brain swelling, a CSF production/absorption mismatch, and cranial venous outflow obstruction (24). Among these, obstruction to cranial venous outflow has received the most attention, venous sinus thrombosis, for example, being a well-known mimic (25), and recently, it has become clear that stenotic lesions on both transverse sinuses in this condition are usual (26, 27). Yet, the extent to which these lesions represent the cause or effect of raised intracranial pressure is uncertain, even if stenting them seems to bring clinical benefit (28–30). Pertinent to the case we describe, however, instances of intracranial hypertension have now been reported, clinically indistinguishable from IIH, which seem to be caused by obstruction to cranial venous outflow in the neck. In these cases, such ambiguity over cause and effect is absent (17, 31–33).

Extracranial venous outflow obstruction has also been implicated in cases of spontaneous intracranial hypotension (18, 34). This condition, caused by a CSF leak, appears to develop spontaneously or following minimal trauma, giving symptoms that cover a spectrum of severity from postural headache, fatigue, dizziness, nausea, vomiting, blurred vision, and light and noise sensitivity, to enforced recumbency, cognitive disturbance, dementia, and even coma (9). The cause of these multiple symptoms is thought to rest largely in the distortion of brain structures that occur as a result of CSF depletion. Spontaneous intracranial hypotension, however, also has its perplexing features: the typical phenotype may not be accompanied by any radiological abnormality, and in some patients, intracranial pressure may be normal or even elevated (8–10).

This last observation, in particular, is difficult to reconcile with the historical view of spontaneous intracranial hypotension, defined partly by intracranial pressures less than 6 cm H_2_0. However, the observation that the closure of a leak in spontaneous intracranial hypotension can lead to rebound intracranial hypertension (35, 36) and the knowledge that CSF leaks may accompany IIH (6–8) imply that spontaneous intracranial hypotension can often represent a complication of IIH, developing when the dura gives way to a weak point under the stress of prolonged elevation of intracranial pressure. In these circumstances, it might not be expected that CSF pressures would fall to very low levels.

This concept, however, while establishing a plausible connection between IIH and spontaneous intracranial hypotension, does not fit well with the idea that IIH is caused by a mismatch between CSF production and CSF absorption since, in this situation, one would expect the complication to effect a cure. On the other hand, it is an encouragement to return to the idea of an external force, such as venous hypertension, acting to elevate intracranial pressure and, recently, two cases of spontaneous intracranial hypotension have been published, caused by narrowing of the jugular veins (18, 34). In both cases, the clinical syndrome was severe, and the radiological findings were striking. In one CSF, the pressure was shown to be in the high normal range (34). Both responded to relief of jugular obstruction with complete resolution of symptoms. Similar syndromes and responses to treatment have been recorded after iatrogenic occlusion of the sigmoid sinus during posterior fossa surgery (37).

**Figure 4:**
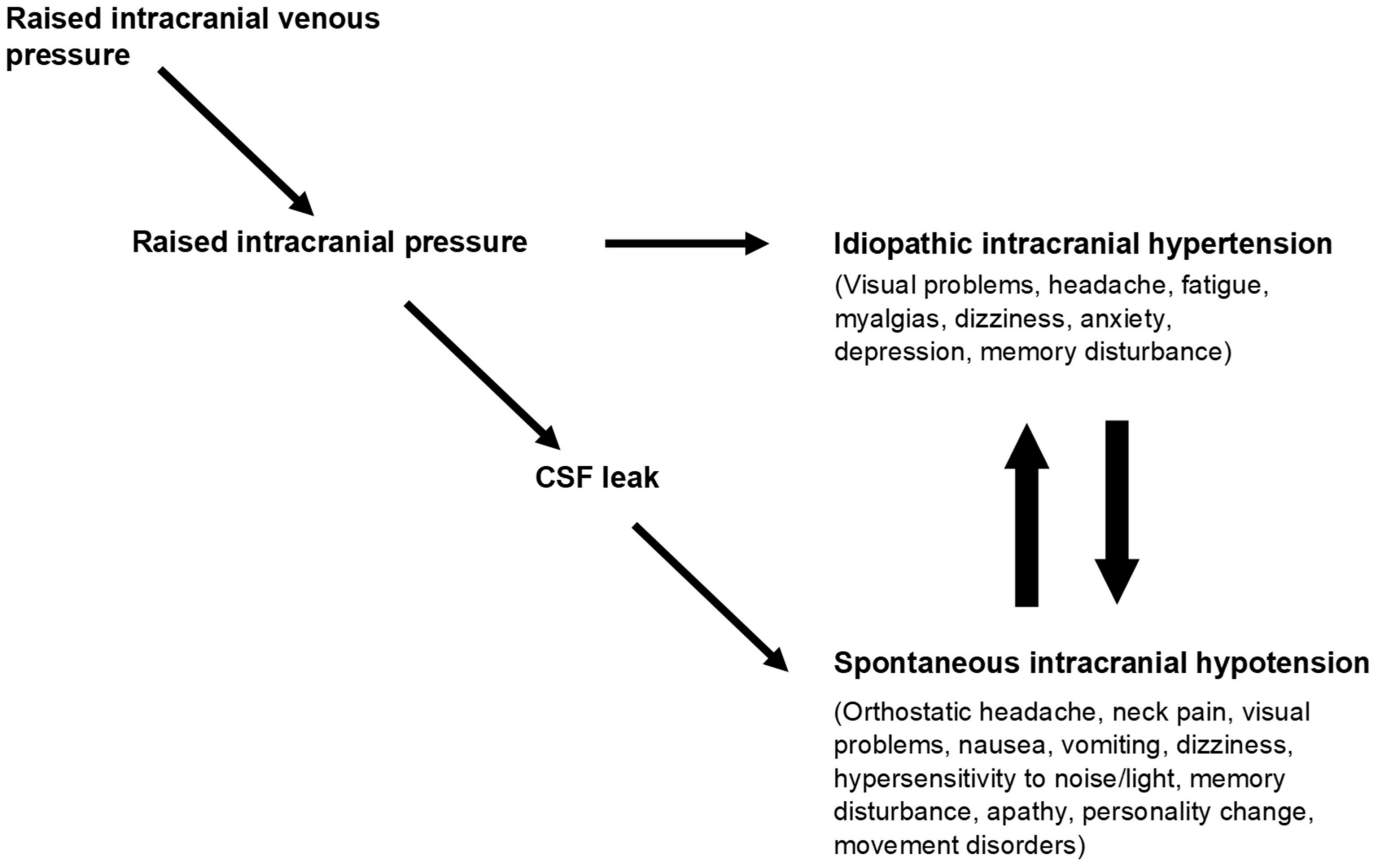
Obstruction to cranial venous outflow causes a rise in intracranial venous pressures leading to a rise in intracranial pressure and the syndrome of IIH. If a CSF leak develops before IIH becomes evident, then the physiological disturbance manifests as spontaneous intracranial hypotension (SIH). IIH and SIH have multiple overlapping symptoms, and patients may reach an equilibrium position between them or may cycle between one and the other, reflecting opposing forces on intracranial pressure. Taken from “A paradigm for chronic fatigue syndrome: caught between idiopathic intracranial hypertension and spontaneous intracranial hypotension; caused by cranial venous outflow obstruction” by J Nicholas P. Higgins & John D. Pickard. Fatigue: Biomedicine, Health & Behavior 2021, 9:3, copyright © 2021 ACFS/ME reprinted by permission of Informa UK Limited, trading as Taylor & Francis Group https://www.tandfonline.com on behalf of ACFS/ME.

Thus, extracranial venous outflow obstruction can give rise to syndromes in which intracranial pressure may be high, normal, or low, depending on the severity of venous obstruction and the presence or absence of a CSF leak. The clinical manifestation of these syndromes is likely to be complex, reflecting the effect of venous obstruction on cerebral perfusion, the effect of abnormal intracranial pressure on brain function and pain-sensitive structures, and the product of distortion of intracranial contents if there is CSF depletion. Importantly, the absolute value of intracranial pressure in these circumstances may be an unreliable indicator of the level of physiological disturbance (16) (Figure 4).

The case we described here conformed to this template. CSF opening pressure was in the normal range (14 cm H_2_O). Her response to CSF drainage was complex, but there were features that suggested that she gained benefit when CSF pressure was reduced. There was mild intracranial venous hypertension, with sagittal sinus pressures of 20 cm H_2_O, this implying a reversal of the normal pressure gradient between CSF and the venous sinuses, seen only when there is a CSF leak or a functioning CSF shunt. She responded positively to venoplasty of the narrowed segments identified on CT and catheter venography, demonstrating the potential reversibility of her clinical syndrome and suggesting targets for treatment. The treatment, itself, while by no means affecting a cure, has been transforming.

What does this mean? Observer bias cannot be discounted in a report without a control group, nor can the effect of placebo, especially when the treatment offered is based on an organic model of disease sympathetic to the widely held view among these patients that they have an illness with a physical cause. Nevertheless, the subtle clinical response to the diagnostic investigations witnessed in this case, the nuanced and incremental response to successive interventions (reminiscent of the response in other similar cases) (18, 34), durable over the course of long follow-up, and easily reconciled with the limited success of the interventions themselves, would require a degree of sophistication and consistency in placebo effect that would be hard to credit.

Equally, it should not be surprising that whatever pathological process is behind IIH, creating a spectrum of increasing clinical severity above a threshold value of 25 cm H_2_O, should also furnish a population of patients with an equivalent illness that does not make the threshold. The requirement for a threshold declares the presence of symptoms in these patients. The key questions are what form these symptoms would take and what diagnostic label might be attached to them. In answer to the first, the form will be contributed from the multiple symptoms seen in IIH, outside those attributed to papilloedema but including those that might be attributable to a CSF leak. In answer to the second, the prevalence of fatigue among these symptoms should make a diagnosis of chronic fatigue syndrome prominent.

The outcome reported here, therefore, which is consistent with another reported previously (38) and with results emerging from recent research (2–5), suggests that investigating patients with chronic fatigue syndrome as if they had a disorder of intracranial pressure might not only bring clinical benefit to individual patients but also contribute to a better understanding of IIH itself and its relationship with spontaneous intracranial hypotension.

## Data availability statement

The original contributions presented in the study are included in the article/supplementary material, further inquiries can be directed to the corresponding author.

## Ethics statement

Ethical review and approval was not required for the study on human participants in accordance with the local legislation and institutional requirements. Written informed consent for participation was not required for this study in accordance with the national legislation and the institutional requirements. Written informed consent was obtained from the individual(s) for the publication of any potentially identifiable images or data included in this article.

## Author contributions

JH advocated the management approach, directed the diagnostic procedures, and wrote the first draft of the manuscript. PA performed the surgical procedures. AL conducted the preliminary clinical assessments and follow-up. All authors reviewed and contributed to the final draft of the manuscript.
